# *Mucuna pruriens* var. *pruriens* Ethanolic Seed Extract Enhances Erectile Function-Related Mechanisms: Integrated In Vitro and In Vivo Evidence

**DOI:** 10.3390/biology15161348

**Published:** 2026-08-09

**Authors:** Supaporn Intatham, Phraepakaporn Kunnaja, Thunyatorn Yimsoo, Mingkwan Na Takuathung, Parirat Khonsung, Kanjana Jaijoy, Sunee Chansakaow, Ratchadawan Puangpradab, Seewaboon Sireeratawong

**Affiliations:** 1 Clinical Research Center for Food and Herbal Product Trials and Development (CR-FAH), Faculty of Medicine, Chiang Mai University, Chiang Mai 50200, Thailand; intatham_s@outlook.com (S.I.); mingkwan.n@cmu.ac.th (M.N.T.); 2 Department of Pharmacology, Faculty of Medicine, Chiang Mai University, Chiang Mai 50200, Thailand; wparirat@hotmail.com; 3 Department of Medical Technology, Faculty of Associated Medical Sciences, Chiang Mai University, Chiang Mai 50200, Thailand; phraepakaporn.k@cmu.ac.th; 4 Laboratory Animal Center, Office of Advanced Science and Technology, Thammasat University, Pathum Thani 12120, Thailand; tyimsoo@tu.ac.th; 5 McCormick Faculty of Nursing, Payap University, Chiang Mai 50000, Thailand; joi.kanjana@gmail.com; 6 Department of Pharmaceutical Sciences, Faculty of Pharmacy, Chiang Mai University, Chiang Mai 50200, Thailand; sunee.c@cmu.ac.th; 7 Queen Sirikit Botanic Garden, The Botanical Garden Organization, Chiang Mai 50180, Thailand; ratchadawanp@hotmail.com

**Keywords:** *Mucuna pruriens*, erectile dysfunction, nitric oxide, cyclic guanosine monophosphate, phosphodiesterase type 5, mounting frequency

## Abstract

*Mucuna pruriens* (Linn.) DC. var. *pruriens* is used as a traditional medication to enhance male sexual function, yet integrated evidence linking vascular-cell mechanisms to in vivo efficacy and safety of a standardized extract remains limited. This study evaluated the ethanolic seed extract of *M. pruriens* var. *pruriens* (MPSE) on erectile function-related mechanisms, addressing the nitric oxide (NO)–cyclic guanosine monophosphate (cGMP)–phosphodiesterase type 5 (PDE5) pathway in vitro, together with mounting frequency, anxiety-like behavior, locomotor activity, and cardiovascular safety in vivo. Soxhlet extraction with 80% ethanol yielded a dark brown, syrupy extract (12.28% *w*/*w*), which was standardized by high-performance thin-layer chromatography to contain 278 mg/g extract (27.8% *w*/*w*) of L-DOPA (Rf 0.38). Mechanistic effects were assessed in human umbilical vein endothelial cells (HUVECs), pulmonary artery smooth muscle cells (PASMCs), and a PDE5A1 enzyme assay. Male Wistar rats received MPSE (50, 100, and 200 mg/kg) orally for 15 days, with hemodynamic assessment in normotensive and Nω-nitro-L-arginine methyl ester (L-NAME)-induced hypertensive rats. MPSE significantly increased eNOS expression and NO production in HUVECs, elevated intracellular cGMP in PASMCs, and directly inhibited PDE5A1 enzymatic activity. Repeated administration significantly increased mounting frequency relative to baseline without altering serum testosterone levels, anxiety-like behavior, locomotor activity, mean arterial pressure, or heart rate. These findings indicate that MPSE modulates erectile function-related mechanisms, primarily through peripheral vascular effects on the NO–cGMP–PDE5 pathway, and suggest a favorable safety profile. Erectile function itself was not directly assessed and warrants confirmation in models of erectile response. MPSE nevertheless represents a promising plant-derived candidate for erectile dysfunction.

## Simple Summary

Erectile dysfunction is a common problem, especially among older men, and it can affect confidence, relationships, and quality of life. Effective medicines exist, but they can be costly, cause side effects, and cannot be taken safely by everyone, particularly some men with heart conditions. This has encouraged the search for gentler, plant-based options. Seeds of the velvet bean (*Mucuna pruriens*), a plant long used in traditional medicine, have been valued for centuries to support male sexual health. In this study, we prepared an extract from these seeds and tested how it works, using human blood vessel cells grown in the laboratory and then male rats. In these blood vessel cells, the extract strengthened the natural chemical signals that help blood vessels relax, a process essential for a normal erection. In rats, it increased male sexual activity without raising testosterone levels, and did not cause anxiety, alter normal movement, or disturb blood pressure or heart rate, even in animals with high blood pressure. These findings suggest the extract acts mainly on the blood vessel signals that support erection, rather than through hormones or the brain, and that it has a favorable safety profile. This study examined the processes that make an erection possible rather than measuring erectile function itself, so more work is still needed. Even so, the extract shows promise as a natural candidate for the management of erectile dysfunction.

## 1. Introduction

Erectile dysfunction is a common male sexual disorder, particularly prevalent among middle-aged and older men, and represents an important clinical and public health concern worldwide [1]. Its pathogenesis is multifactorial and involves the complex interplay of vascular, neurogenic, hormonal, psychological, and anatomical factors that collectively impair erectile function [2]. Prevalence increases markedly with age. A United States population-based cross-sectional survey conducted in 2021 reported that 62.5% of men aged 65 years and older met the International Index of Erectile Function (IIEF-5) criteria for erectile dysfunction [3], whereas a Thai population-based survey conducted in 2008 among men aged 40–70 years reported a prevalence of 42.18% [4]. Erectile dysfunction is also strongly associated with cardiometabolic risk conditions, including hypertension, obesity, dyslipidemia, diabetes mellitus, and cardiovascular disease, which contribute to endothelial dysfunction and impaired penile hemodynamics [5,6]. Beyond its physiological consequences, the condition imposes a substantial psychosocial burden, including diminished self-confidence, emotional distress, interpersonal strain, and a marked decline in overall quality of life [7,8,9,10,11]. Together, these findings underscore the clinical burden of erectile dysfunction and the need for effective therapeutic strategies.

A central pathophysiological feature in many cases of erectile dysfunction is endothelial and cavernosal smooth muscle dysfunction, which disrupts the hemodynamic mechanisms required for penile erection [12]. Nitric oxide (NO), released from non-adrenergic non-cholinergic neurons and synthesized by endothelial nitric oxide synthase (eNOS), diffuses into cavernosal smooth muscle cells and activates soluble guanylyl cyclase, promoting the conversion of guanosine triphosphate (GTP) to cyclic guanosine monophosphate (cGMP). Increased intracellular cGMP reduces calcium levels, leading to smooth muscle relaxation, enhanced arterial inflow, and penile erection [12,13]. Intracellular cGMP concentrations are regulated by phosphodiesterase type 5 (PDE5), which hydrolyzes cGMP to inactive 5′-guanosine monophosphate (5′-GMP), thereby terminating its effect [14]. Collectively, these mechanisms establish the NO–cGMP–PDE5 pathway as a central regulatory axis in erectile physiology and the primary pharmacological target in contemporary treatment for erectile dysfunction [15,16]. In experimental studies of sexual behavior, changes in copulatory parameters may be influenced not only by erectile function-related mechanisms but also by psychological factors such as anxiety and changes in general locomotor activity [17]. Anxiety is known to suppress erectile responses through increased sympathetic activity and central modulation of sexual arousal [18,19], whereas increased locomotor activity may nonspecifically enhance behavioral performance by facilitating copulatory parameters independent of erectile function-related mechanisms [20]. Evaluation of anxiety-like behavior and locomotor activity is therefore important when investigating candidate agents for erectile dysfunction, to distinguish central behavioral influences from direct effects on erectile function-related pathways.

Oral PDE5 inhibitors such as sildenafil are considered the first-line pharmacological therapy for erectile dysfunction and are widely used because of their proven efficacy and convenience [5]. These agents inhibit the PDE5 enzyme in penile tissue, thereby preventing the degradation of cGMP and promoting smooth muscle relaxation in the corpus cavernosum [14,21]. Their use is nevertheless contraindicated in patients receiving nitrate-containing medications, in whom additive vasodilatory effects may lead to severe and potentially life-threatening hypotension [5,22]. Alternative options such as intraurethral alprostadil are used less frequently because of their relatively complex administration and potential adverse effects, including penile pain [5,23], and treatment cost together with drug-related side effects may further limit long-term use [24,25]. There remains, therefore, a continuing need to explore alternative therapeutic approaches for the management of erectile dysfunction.

*M. pruriens* (L.) DC., known as *Kapikacchu* in Ayurvedic medicine and as *Mha-Mui* in Thailand, is traditionally classified as a *Vrishya* (aphrodisiac) herb and has been used for centuries to enhance sexual performance and improve male reproductive health [26]. Among its varieties, *M. pruriens* var. *pruriens* is characterized by the presence of irritant hairs on the pods, which can cause allergic or irritative reactions upon contact [27]. Seed extracts of this variety have been shown to enhance mating behavior and to ameliorate ethanol-induced reductions in sperm quality and sperm production in male rats [28], and to improve sexual performance while restoring testosterone levels and reproductive protein expression in chronic unpredictable mild stress male mice [29]. Two gaps nevertheless remain. First, a recent comprehensive review concluded that variability in plant parts, extraction procedures, and the extent of phytochemical characterization restricts meaningful comparison across published studies, and recommended the use of standardized seed extracts with defined chemical markers in future pharmacological investigations [30]. Second, previous studies have examined either cellular mechanisms or behavioral outcomes in isolation, so that the vascular-cell actions of a standardized extract of *M. pruriens* var. *pruriens* have not been linked to its in vivo behavioral efficacy and cardiovascular safety within a single study. Thus, the present study was designed to address these gaps by evaluating a chemically standardized ethanolic seed extract of *M. pruriens* var. *pruriens* (MPSE) in an integrated experimental framework. Modulation of the NO–cGMP–PDE5 pathway was examined in primary human vascular cells and in a PDE5A1 enzyme assay, and mounting frequency, serum testosterone levels, anxiety-like behavior, locomotor activity, and cardiovascular safety under normotensive and hypertensive conditions were assessed in vivo.

## 2. Materials and Methods

### 2.1. Preparation of MPSE

#### 2.1.1. Raw Material Preparation and Quality Control of Raw Material

Roasted seeds of *M. pruriens* var. *pruriens* were obtained from Sao Hai District, Saraburi Province, Thailand. Species identification was performed by comparison with reference samples collected in 2017, which were kept at the Medicinal Plant Innovation Center, Faculty of Pharmacy, Chiang Mai University (MPP 01-05).

Quality control was performed according to Thai Herbal Pharmacopoeia (THP) standards [31]. Physicochemical analysis encompassed loss on drying, total ash, acid-insoluble ash, and extractive values (95% ethanol and chloroform-saturated water). Thin-layer chromatography (TLC) profiling was conducted on Silica gel GF_254_ plates using *n*-butanol:acetic acid:water (4:1:1) as the mobile phase. Detection was achieved using UV light (254 and 366 nm) and spraying with the ninhydrin reagent.

#### 2.1.2. Preparation of the Plant Extract

Seed powder of *M. pruriens* var. *pruriens* was extracted with 80% ethanol (1:6 *w*/*v*) using a Soxhlet apparatus for three cycles of 8 h each. After extraction, the solvent was removed by rotary evaporation, yielding a dark brown, syrupy concentrate, which was stored in an amber glass bottle under refrigeration.

#### 2.1.3. Phytochemical Analysis of the Extract by High-Performance Thin-Layer Chromatography (HPTLC)

##### Preparation of Standard and Test Solutions

A standard stock solution of L-DOPA (2 mg/mL) was prepared in 80% ethanol and diluted to produce a calibration curve ranging from 0.9 to 1.9 mg/mL. The standard solution was filtered using a 0.45 μm nylon syringe filter. Test samples were prepared at 5 mg/mL in 80% ethanol and filtered through a 0.22 μm nylon syringe filter prior to injection.

##### Chromatographic Conditions

Qualitative and quantitative analyses were conducted using a CAMAG HPTLC system. Separation was achieved on Silica gel GF_254_ plates (20 ×10 cm; Merck, Darmstadt, Germany). Samples and standards were applied as 8 mm bands with a precise applicator, positioned at an initial Y-axis of 15 mm, a first track X-axis of 15 mm, and a 12 mm distance between tracks. Chromatographic development utilized a mobile phase of *n*-butanol, acetic acid, and water (4:1:1, *v*/*v*/*v*), with the solvent front migrating 70 mm. After development, plates were analyzed using an HPTLC Densitometer (CAMAG, Muttenz, Switzerland). Densitometric scanning was performed in absorbance mode at 280 nm. Compound identification and quantification were accomplished by comparing the absorbance and spectral characteristics of sample bands to those of standard solutions.

### 2.2. In Vitro Evaluation of MPSE on the NO–cGMP–PDE5 Pathway

#### 2.2.1. Cell Culture

Primary human umbilical vein endothelial cells (HUVEC; pooled, normal) were purchased from the American Type Culture Collection (ATCC, Manassas, VA, USA) and cultured in Vascular Cell Basal Medium (ATCC PCS-100-030, Manassas, VA, USA) supplemented with the Endothelial Cell Growth Kit-BBE (ATCC PCS-100-040), which contains bovine brain extract, recombinant human epidermal growth factor (rhEGF), L-glutamine, heparin sulfate, hydrocortisone hemisuccinate, fetal bovine serum (FBS), and ascorbic acid. Primary human pulmonary artery smooth muscle cells (PASMC; normal) were also obtained from ATCC and cultured in Vascular Cell Basal Medium (ATCC PCS-100-030) supplemented with the Vascular Smooth Muscle Cell Growth Kit (ATCC PCS-100-042, Manassas, VA, USA), containing recombinant human fibroblast growth factor-β (rhFGF-b), recombinant human insulin, ascorbic acid, L-glutamine, rhEGF, and FBS. HUVECs and PASMCs were maintained in a humidified incubator at 37 °C with 5% CO_2_. Cells were subcultured at approximately 80–90% confluence and used at passages 3–6 for all subsequent experiments.

#### 2.2.2. Cell Viability Assay

A cytotoxicity assay was performed using the MTT assay to determine the non-toxic concentrations of MPSE, sildenafil, and Nω-nitro-L-arginine methyl ester (L-NAME) as a non-selective nitric oxide synthase (NOS) inhibitor prior to mechanistic studies. The selected concentrations were subsequently used for further experiments. Briefly, HUVECs and PASMCs were seeded in 96-well plates at a density of 1.0 × 10^4^ cells/well and allowed to attach overnight. The cells were then treated with various concentrations of MPSE, sildenafil, and L-NAME for 24 h. Subsequently, MTT solution (0.5 mg/mL) was added and incubated for 3 h at 37 °C. The resulting formazan crystals were dissolved in DMSO, and absorbance was measured at 570 nm using a microplate reader. Cell viability was expressed as a percentage relative to the control group.

#### 2.2.3. Determination of eNOS mRNA Expression in HUVECs

HUVECs were seeded in 6-well plates at a density of 1.5 × 10^5^ cells/well and allowed to attach overnight under standard culture conditions. After reaching approximately 70–80% confluence, the culture medium was replaced with fresh medium containing MPSE (50, 100, and 200 µg/mL), sildenafil (25 µM), or L-NAME (100 µM). Untreated cells cultured in complete medium served as the control group. Cells were incubated with the test compounds for 24 h. After treatment, the culture medium was removed, and the cells were gently washed twice with cold phosphate-buffered saline (PBS). Cells were then lysed directly in the culture wells using the lysis buffer provided in the FavorPrep™ Blood/Cultured Cell Total RNA Mini Kit (Favorgen Biotech Corp., Ping-Tung, Taiwan) and harvested by scraping. The lysates were transferred into RNase-free microcentrifuge tubes for total RNA extraction according to the manufacturer’s instructions. RNA concentration and purity were determined spectrophotometrically using a NanoDrop instrument (Thermo Fisher Scientific, Waltham, MA, USA) by measuring absorbance at 260 and 280 nm. Only RNA samples with an A260/A280 ratio greater than 2.0 were used for subsequent analysis. Complementary DNA (cDNA) was synthesized from total RNA using ReverTra Ace™ qPCR RT Master Mix with gDNA Remover (TOYOBO Co., Ltd., Osaka, Japan). Briefly, RNA samples were first treated with gDNA remover at 37 °C for 5 min to eliminate genomic DNA contamination, followed by reverse transcription at 37 °C for 15 min and 50 °C for 5 min, with enzyme inactivation at 98 °C for 5 min. Quantitative real-time PCR (RT-qPCR) was performed using PerfeCTa SYBR Green FastMix, Low ROX (Quantabio, LLC, Beverly, MA, USA). Gene-specific primers for *Homo sapiens* nitric oxide synthase 3 (NOS3; eNOS) and the housekeeping gene glyceraldehyde-3-phosphate dehydrogenase (GAPDH) were designed using an online primer design program. Amplification was performed under the following conditions: initial polymerase activation at 95 °C for 3 min, followed by 40 cycles of denaturation at 95 °C for 15 s, annealing at 58 °C for 15 s, and extension at 72 °C for 30 s. Melt curve analysis was conducted to confirm amplification specificity. Relative mRNA expression levels of eNOS were normalized to GAPDH expression and calculated using the comparative Ct (2^−ΔΔCt^) method.

#### 2.2.4. Determination of NO Production in HUVECs

HUVECs were seeded in 96-well plates at a density of 1.0 × 10^4^ cells/well and allowed to attach overnight under standard culture conditions. The cells were then treated with MPSE (50, 100, and 200 µg/mL), sildenafil (25 µM), or L-NAME (100 µM) for 24 h, while untreated cells served as the control group. After treatment, culture supernatants were carefully collected and centrifuged at 3000× *g* for 10 min to remove cell debris. The clarified supernatants were then used to determine nitrite levels, an indicator of NO production, using the Griess reaction. NO production in HUVECs was determined by measuring nitrite levels in the culture supernatant using the Griess reaction, as nitrite represents a stable oxidized metabolite of NO. Culture supernatants were mixed with an equal volume of Griess reagent consisting of 0.1% N-(1-naphthyl)ethylenediamine dihydrochloride, 1% sulfanilamide, and 2.5% phosphoric acid (Sigma-Aldrich, St. Louis, MO, USA). The reaction mixture was incubated at room temperature for 10 min, and the absorbance was measured at 540 nm using a microplate reader. Nitrite concentrations in the samples were calculated from a standard calibration curve generated using sodium nitrite (Sigma-Aldrich, St. Louis, MO, USA) in the range of 1–100 µM prepared in the same manner as the samples.

#### 2.2.5. Measurement of Intracellular cGMP Levels in PASMCs

PASMCs were seeded in 6-well plates at a density of 3.0 × 10^5^ cells/well and cultured overnight to allow cell attachment under standard culture conditions. Cells were subsequently treated with MPSE (50, 100, and 200 µg/mL), sildenafil (25 µM), or L-NAME (100 µM) for 24 h, while untreated cells served as the control group. After treatment, cells were washed twice with cold PBS to remove residual medium and lysed using Cell Lysis Buffer 5 (diluted 1:5) provided in the Parameter™ cGMP Competitive ELISA Kit (R&D Systems, Minneapolis, MN, USA). The cell lysates were collected by scraping and transferred into microcentrifuge tubes for intracellular cGMP determination according to the manufacturer’s instructions. Intracellular cGMP levels in PASMCs were measured using a competitive ELISA assay. Briefly, PASMC lysates were added to ELISA plates together with standards and controls. After incubation with cGMP conjugate and primary antibody for 3 h at room temperature on a horizontal orbital microplate shaker (500 ± 50 rpm), the plates were washed and incubated with substrate solution for 30 min in the dark. The reaction was terminated by adding the stop solution, and absorbance was measured at 450 nm with wavelength correction at 570 nm using a microplate reader. Intracellular cGMP concentrations were calculated from the standard curve according to the manufacturer’s protocol.

#### 2.2.6. Measurement of PDE5 Inhibitory Activity

PDE5 isoform A1 (PDE5A1) inhibitory activity was determined using a fluorescence polarization–based assay kit (BPS Bioscience Inc., San Diego, CA, USA) according to the manufacturer’s instructions. Briefly, FAM-Cyclic-3′,5′-GMP substrate was incubated with PDE5A1 enzyme in the presence or absence of MPSE. A buffer-only control (negative control) was included, in which the enzyme reaction mixture contained buffer without any inhibitor. Sildenafil was used as a positive control inhibitor. After incubation at room temperature for 1 h, the binding agent solution was added, and the mixture was further incubated for 30 min. Fluorescence polarization was measured using a microplate reader with excitation at 475–495 nm and emission at 518–538 nm. PDE5 inhibitory activity was calculated according to the manufacturer’s protocol after subtraction of blank values.

### 2.3. In Vivo Evaluation of MPSE on Mounting Behavior and Safety Profile

#### 2.3.1. Animals, Housing Conditions, and Ethical Approval

Male and female Wistar rats (10 weeks old) were obtained from the National Laboratory Animal Center, Mahidol University, Nakhon Pathom, Thailand. The animals were housed in a controlled experimental animal facility under standard environmental conditions at a temperature of 25 ± 1 °C, relative humidity of approximately 60%, and a 12 h light/dark cycle. Rats were provided with standard laboratory chow and drinking water ad libitum. All animals were acclimatized for at least 1 week prior to the start of the experiment. All experimental procedures were reviewed and approved by the Animal Ethical Committee of the Faculty of Medicine, Chiang Mai University (Approval No. 39/2559; approved on 7 December 2016). The sample size of six animals per group was determined a priori using G*Power version 3.1.9.2 (Heinrich-Heine-Universität Düsseldorf, Düsseldorf, Germany) and was specified in the approved protocol.

#### 2.3.2. Evaluation of Mounting Frequency and Serum Testosterone Levels

##### Preparation of Ovariectomized Hormone-Primed Female Rats

Female Wistar rats (80 days old; n = 30) were bilaterally ovariectomized under aseptic conditions to eliminate the influence of endogenous ovarian hormones. Briefly, animals were anesthetized, and small dorsolateral skin incisions were made on both flanks. The ovaries were exteriorized through the muscle wall, ligated at the oviduct, and excised. The muscle layer was closed with absorbable sutures, and the skin was closed with wound clips. Animals were allowed to recover for at least 30 days before hormonal priming to ensure depletion of endogenous ovarian hormones. To standardize hormonal conditions, the ovariectomized females were hormonally primed with estrogen (2 µg/kg body weight) administered 48 h before the experiment, followed by progesterone (500 µg/kg body weight) administered 4–6 h before mounting frequency assessment. The hormone-primed females were then introduced into the cages of male rats to evaluate mounting frequency.

##### Assessment of Mounting Frequency and Measurement of Serum Testosterone Levels

Male rats were initially trained to screen for mounting behavior by introducing each animal to an ovariectomized, hormone-primed female in a transparent observation cage for 5 min. Males that exhibited mounting behavior were considered sexually active and selected for further experiments. A total of 30 qualified male rats were randomly assigned into five groups (n = 6 per group) as follows: Group 1 (control group) received distilled water (2 mL/kg body weight); Group 2 received sildenafil (5 mg/kg body weight); and Groups 3–5 received MPSE at doses of 50, 100, and 200 mg/kg body weight, respectively. All treatments were administered orally once daily for 15 consecutive days. For mounting frequency assessment, each male rat was placed together with an ovariectomized, hormone-primed female rat in a transparent observation cage, and mounting behavior was observed for 2 h. Mounting frequency, defined as the number of mounts performed by the male prior to ejaculation, was recorded. Mounting frequency was assessed before treatment initiation and on days 5, 10, and 15 after treatment. At the end of the experimental period (day 15), the male rats were deeply anesthetized with thiopental sodium (150 mg/kg body weight, i.p.) [32], and blood samples were collected via cardiac puncture. Blood samples were allowed to clot at room temperature and then centrifuged at 3500 rpm for 10 min to obtain serum, which was stored at −20 °C until analysis. Serum testosterone levels were determined using an electrochemiluminescence immunoassay (ECLIA) on a Cobas e411 analyzer (Roche Diagnostics, Mannheim, Germany). This assay is based on a competitive immunoassay principle, in which endogenous testosterone competes with a labeled testosterone derivative for binding to a specific antibody. The resulting electrochemiluminescent signal is inversely proportional to the concentration of testosterone in the sample.

#### 2.3.3. Assessment of Anxiety-like Behavior

Thirty-six male rats were randomly assigned into six groups (n = 6 per group) as follows: Group 1 (control group) received distilled water (2 mL/kg body weight); Group 2 received sildenafil (5 mg/kg body weight); Group 3 (positive control group) received diazepam (5 mg/kg body weight); and Groups 4–6 received MPSE at doses of 50, 100, and 200 mg/kg body weight, respectively. All treatments were administered orally once daily for 15 consecutive days. On day 15, 30 min after treatment administration, anxiety-like behavior was assessed using the elevated plus maze test. The elevated plus maze apparatus consisted of two open arms and two closed arms and was elevated approximately 70 cm above the floor. Each rat was placed individually at the center of the maze, facing an open arm, and its behavior was recorded on video for 5 min. The time spent in the open and closed arms was measured and used for behavioral evaluation.

#### 2.3.4. Assessment of Locomotor Activity

Thirty-six male rats were randomly divided into six groups (n = 6 per group) as follows: Group 1 (control group) received distilled water (2 mL/kg body weight); Group 2 received sildenafil (5 mg/kg body weight); Group 3 (positive control group) received diazepam (5 mg/kg body weight); and Groups 4–6 received MPSE at doses of 50, 100, and 200 mg/kg body weight, respectively. All treatments were administered orally once daily for 15 consecutive days. Locomotor activity was evaluated on Day 1 and Day 15, 30 min after treatment administration, using an automated activity monitoring apparatus. Each rat was placed individually in a square Plexiglas chamber (17.5 × 18 × 12 inches) equipped with infrared light-beam sensors that transmitted signals to a data acquisition system for movement detection. Spontaneous locomotor activity, including both horizontal and vertical movements, was recorded at 5-min intervals over a total observation period of 120 min. The sum of horizontal and vertical activity counts was used as an index of locomotor activity to compare the experimental groups.

#### 2.3.5. Assessment of Mean Arterial Pressure and Heart Rate in Normotensive Male Rats

Thirty-six male rats were randomly assigned into six groups (n = 6 per group) as follows: Group 1 (control group) received distilled water (2 mL/kg body weight); Group 2 received sildenafil (5 mg/kg body weight); Group 3 (positive control group) received prazosin (2 mg/kg body weight); and Groups 4–6 received MPSE at doses of 50, 100, and 200 mg/kg body weight, respectively. Mean arterial pressure was measured noninvasively at the tail using a tail-cuff blood pressure monitoring system, Model MRBP System (IITC Life Science, Woodland Hills, CA, USA). Heart rate was recorded simultaneously using the same apparatus. Baseline measurements were obtained prior to treatment administration. All treatments were administered orally, and mean arterial pressure and heart rate were remeasured 30 min after administration.

#### 2.3.6. Assessment of Mean Arterial Pressure and Heart Rate in L-NAME-Induced Hypertensive Male Rats

Mean arterial pressure and heart rate were measured using the same procedure as described for normotensive rats to obtain baseline values prior to hypertension induction. Hypertension was induced in male rats by oral administration of L-NAME at a dose of 50 mg/kg body weight for 2 consecutive days. After the induction period, mean arterial pressure was remeasured, and male rats were considered hypertensive when mean arterial pressure values were significantly higher than their baseline levels (*p* < 0.05). A total of 36 hypertensive male rats were then selected and randomly assigned into six groups (n = 6 per group) as follows: Group 1 (control group) received distilled water (2 mL/kg body weight); Group 2 received sildenafil (5 mg/kg body weight); Group 3 (positive control group) received prazosin (2 mg/kg body weight); and Groups 4–6 received MPSE at doses of 50, 100, and 200 mg/kg body weight, respectively. Mean arterial pressure and heart rate were measured immediately before treatment administration and again 30 min after oral administration.

### 2.4. Statistical Analysis

Data are presented as mean ± S.E.M. For all in vitro experiments, three independent experiments were performed, each with three technical replicates per condition. Technical replicates were averaged within each experiment, and the resulting experimental means were used as the unit of statistical analysis. For the cell viability assay, concentration groups were compared with the untreated control by one-way analysis of variance (ANOVA) followed by Dunnett’s multiple comparison test. For the eNOS mRNA expression, NO production, and intracellular cGMP assays, comparisons among multiple groups were performed using one-way ANOVA followed by Tukey’s multiple comparison test. For the PDE5A1 inhibitory activity assay, percentage inhibition was calculated for each independent experiment relative to the pooled mean signal of the buffer-only control (negative control). Because MPSE and sildenafil were tested using different concentration scales and were not intended for direct comparison with each other, each compound was analyzed separately together with the negative control using one-way ANOVA followed by Tukey’s multiple comparison test.

For the in vivo experiments, normality of the data was assessed using the Shapiro–Wilk test. Because mounting frequency was assessed repeatedly in the same animals, it was analyzed within each group across assessment days using a linear mixed model followed by Bonferroni-corrected pairwise comparisons. Serum testosterone levels were compared among groups using one-way ANOVA followed by Tukey’s multiple comparison test. Elevated plus maze parameters were not normally distributed and were therefore compared among groups using the Kruskal–Wallis test followed by Dunn’s multiple comparison test. Locomotor activity was compared among groups at each 5-min observation interval using one-way ANOVA followed by Tukey’s multiple comparison test. Mean arterial pressure and heart rate were compared among groups at each time point using one-way ANOVA followed by Tukey’s multiple comparison test, and values before and after treatment within the same group were compared using a paired *t*-test. Statistical significance was defined as *p* < 0.05. All statistical analyses were performed using SPSS Statistics version 25 (SPSS Inc., Chicago, IL, USA).

## 3. Results

### 3.1. Preparation of MPSE and Quality Control

The quality of roasted seeds of *M. pruriens* var. *pruriens* was assessed based on physical and chemical properties. The analysis indicated a loss on drying of 0.80 ± 0.01% *w*/*w*, a 95% ethanol-soluble extractive value of 0.20 ± 0.00% *w*/*w*, a water extractive value of 1.17 ± 0.00% *w*/*w*, a total ash content of 3.98 ± 0.01% *w*/*w*, and an acid-insoluble ash content of 0.06 ± 0.01% *w*/*w*. TLC confirmed the presence of L-DOPA in the crude extract of the raw material. Using ninhydrin as a detection reagent (yielding a brown spot), the L-DOPA standard produced an Rf value of 0.38. The *M. pruriens* var. *pruriens* seed extract displayed a spot matching that of L-DOPA. All measured parameters were consistent with the preliminary specifications previously reported [33].

**Figure 1:**
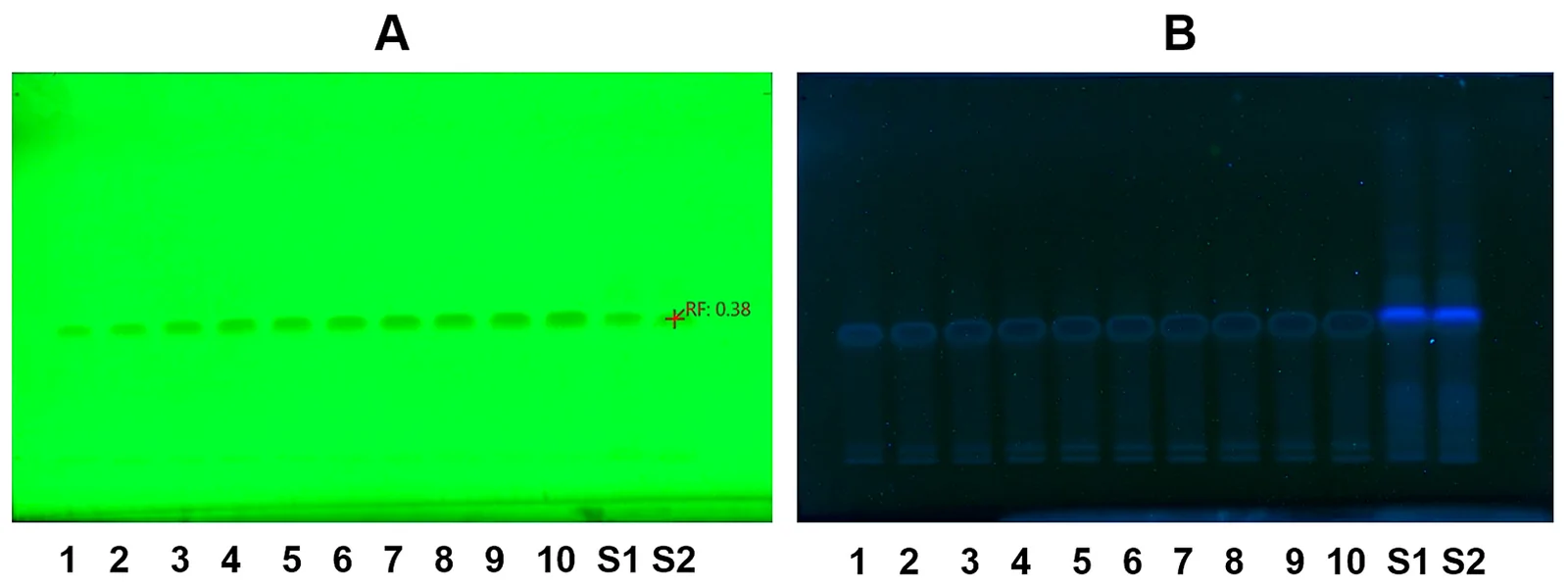
Chromatographic profiles of MPSE. Detected with UV 254 nm (**A**); Detected with UV 366 nm (**B**); spots 1–10 = standard L-DOPA with concentrations ranging from 0.9 to 1.9 mg/mL; S1 and S2 = MPSE. (S1 and S2 indicate technical replicates derived from the same sample batch).

The *M. pruriens* var. *pruriens* seed powder was continuously extracted with 80% ethanol, and the resulting solution was concentrated by rotary evaporation to yield a dark brown syrup (12.28% *w*/*w*). The quality of the extract was subsequently verified using HPTLC analysis. HPTLC analysis of MPSE identified a prominent spot at Rf 0.38, corresponding to the L-DOPA standard. This primary constituent was quantified at a concentration of 1.39 mg/mL. An unidentified spot was observed at Rf 0.42 (clearly visible at 366 nm UV), representing a potential candidate for future characterization and study (Figure 1).

### 3.2. In Vitro Evaluation of MPSE on the NO–cGMP–PDE5 Pathway

#### 3.2.1. Determination of Non-Cytotoxic Concentrations

**Figure 2:**
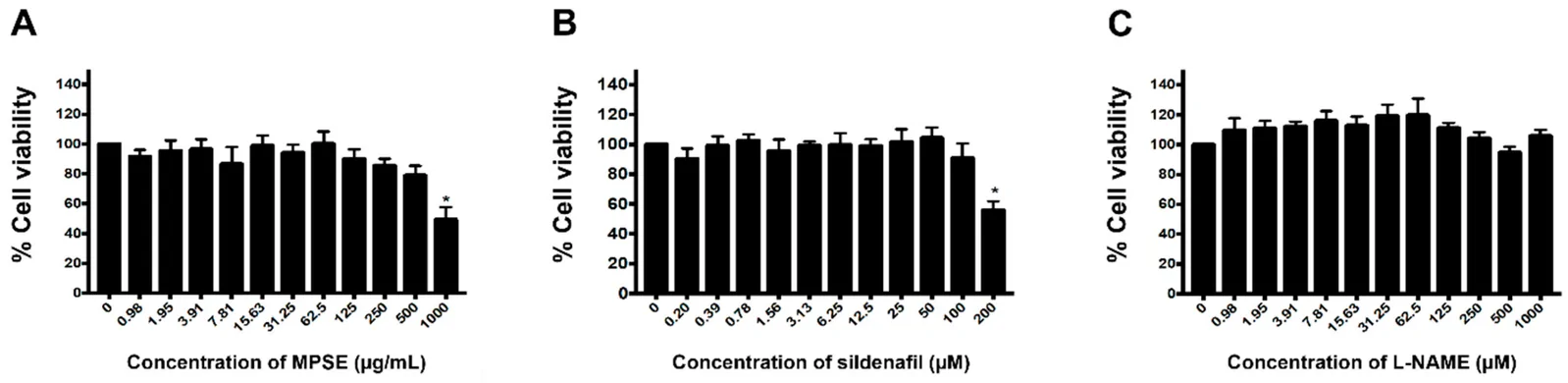
Effects of MPSE (**A**), sildenafil (**B**), and L-NAME (**C**) on HUVEC viability determined by the MTT assay. HUVECs were treated with the indicated concentrations of MPSE, sildenafil, or L-NAME. Data are presented as mean ± S.E.M. (*n* = 3). Concentration groups were compared with the control by one-way ANOVA followed by Dunnett’s multiple comparison test. * *p* < 0.05.

**Figure 3:**
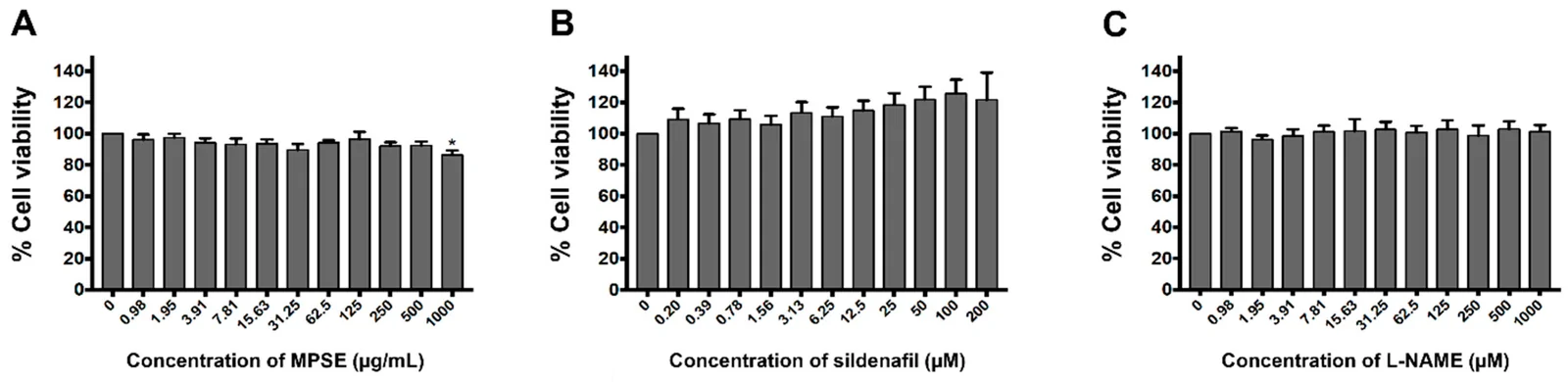
Effects of MPSE (**A**), sildenafil (**B**), and L-NAME (**C**) on PASMC viability determined by the MTT assay. PASMCs were treated with the indicated concentrations of MPSE, sildenafil, or L-NAME. Data are presented as mean ± S.E.M. (*n* = 3). Concentration groups were compared with the control by one-way ANOVA followed by Dunnett’s multiple comparison test. * *p* < 0.05.

The cytotoxic effects of MPSE, sildenafil, and L-NAME on HUVECs and PASMCs were evaluated using the MTT assay. The results showed that MPSE at concentrations of 50, 100, and 200 µg/mL did not significantly affect cell viability in either HUVECs or PASMCs compared with the control groups. Similarly, sildenafil and L-NAME at concentrations of 25 µM and 100 µM, respectively, showed no significant cytotoxic effects on either cell type. Based on these findings, these concentrations were selected for subsequent experiments (Figure 2 and Figure 3).

#### 3.2.2. Effects of MPSE on eNOS mRNA Expression in HUVECs

**Figure 4:**
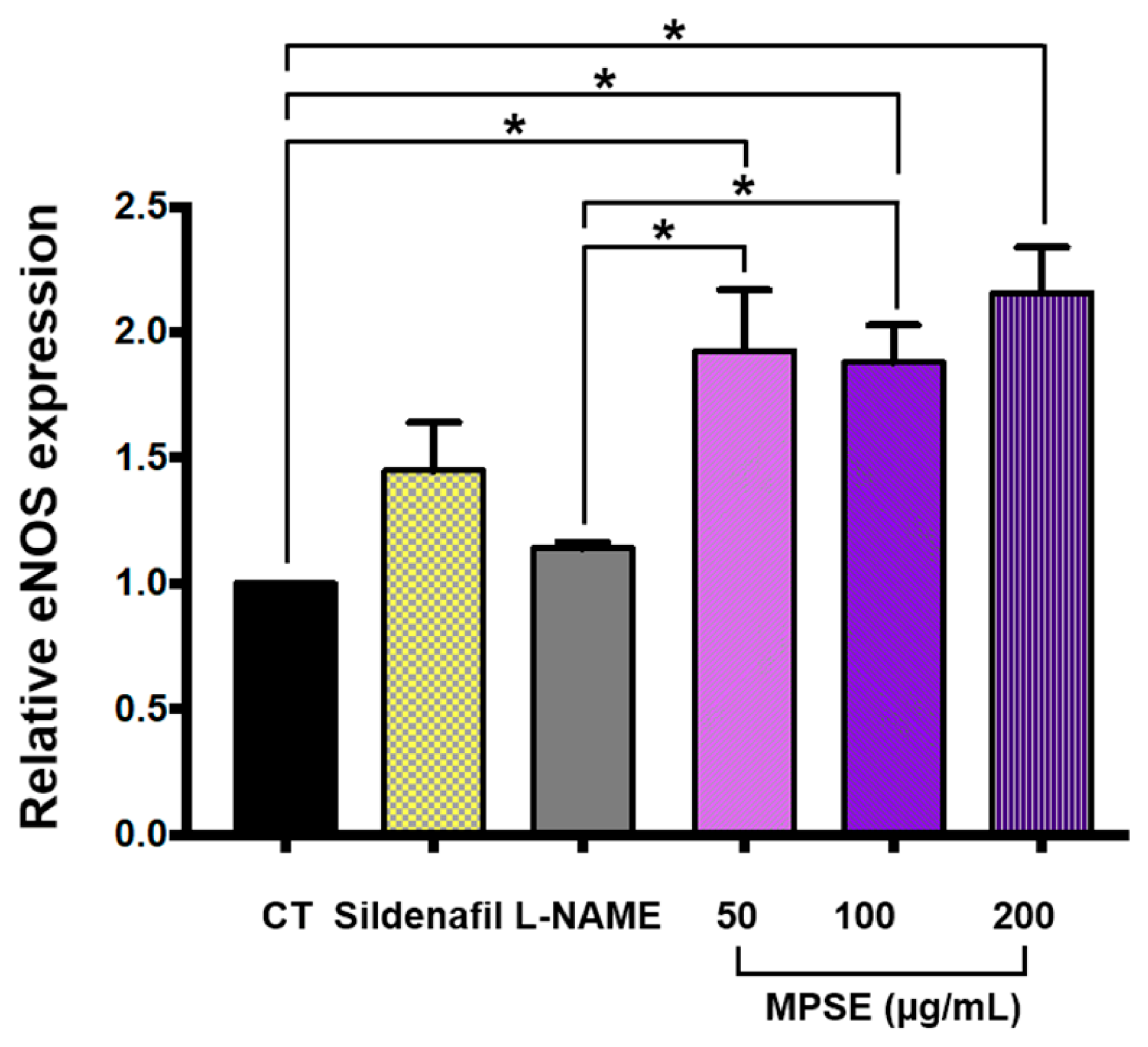
Effect of MPSE on eNOS gene expression in HUVECs. Relative eNOS gene expression was determined by quantitative real-time PCR (RT-qPCR) and normalized to the glyceraldehyde-3-phosphate dehydrogenase (GAPDH) gene. Data are presented as mean ± S.E.M. (*n* = 3). Groups were compared by one-way ANOVA followed by Tukey’s multiple comparison test. * *p* < 0.05 indicates a statistically significant difference between the indicated groups. CT, control group.

MPSE at concentrations of 50, 100, and 200 µg/mL significantly increased eNOS gene expression in HUVECs compared with the control group. Furthermore, MPSE at 50 and 100 µg/mL significantly increased eNOS expression compared with the L-NAME group. In contrast, neither sildenafil nor L-NAME significantly altered eNOS expression relative to the control group (Figure 4).

#### 3.2.3. Effects of MPSE on NO Production in HUVECs

**Figure 5:**
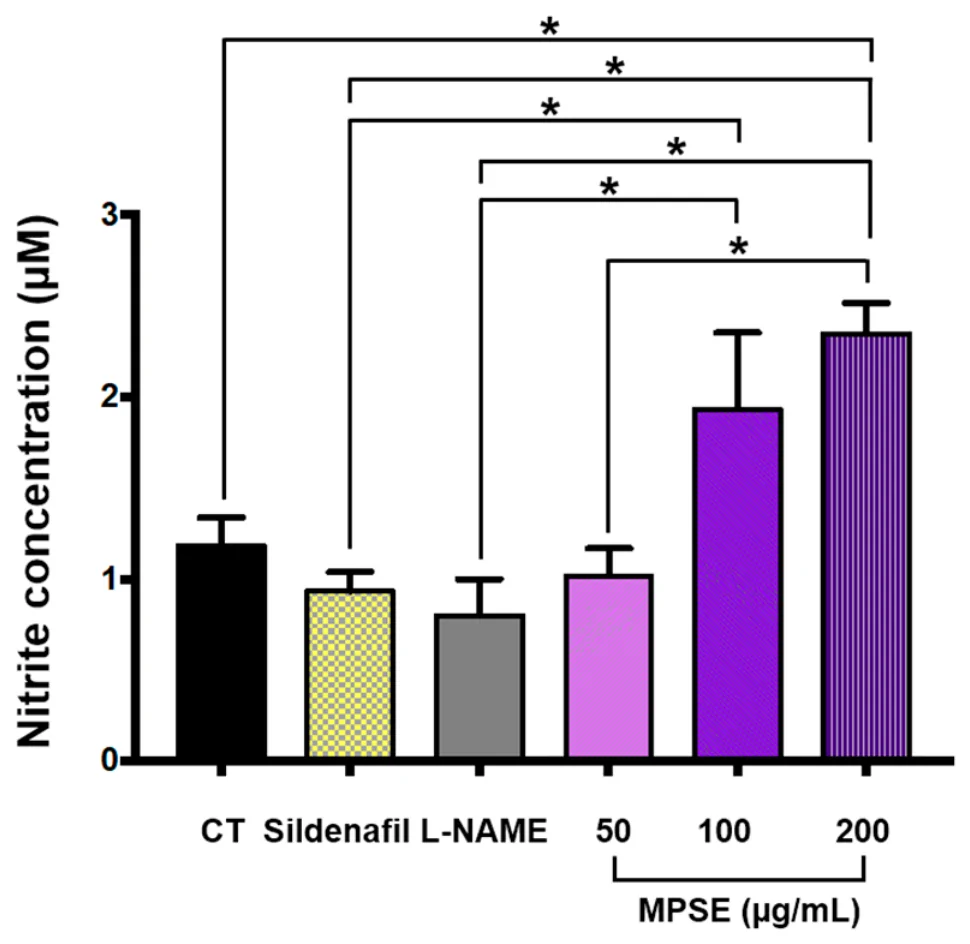
Effect of MPSE on NO production in HUVECs. NO production was determined by measuring nitrite levels in the culture supernatant using the Griess reaction. Data are presented as mean ± S.E.M. (*n* = 3). Groups were compared by one-way ANOVA followed by Tukey’s multiple comparison test. * *p* < 0.05 indicates a statistically significant difference between the indicated groups. CT, control group.

Analysis of NO production in HUVECs revealed that MPSE at 200 µg/mL significantly increased NO production compared with the control group, sildenafil-, and L-NAME-treated groups, as well as the MPSE 50 µg/mL treatment group. In addition, MPSE at 100 µg/mL significantly increased NO production compared with the sildenafil- and L-NAME-treated groups, but did not differ significantly from the control group. The highest level of NO production was observed in the MPSE 200 μg/mL treatment group, which was significantly higher than that of the control group (Figure 5).

#### 3.2.4. Effects of MPSE on Intracellular cGMP Levels in PASMCs

**Figure 6:**
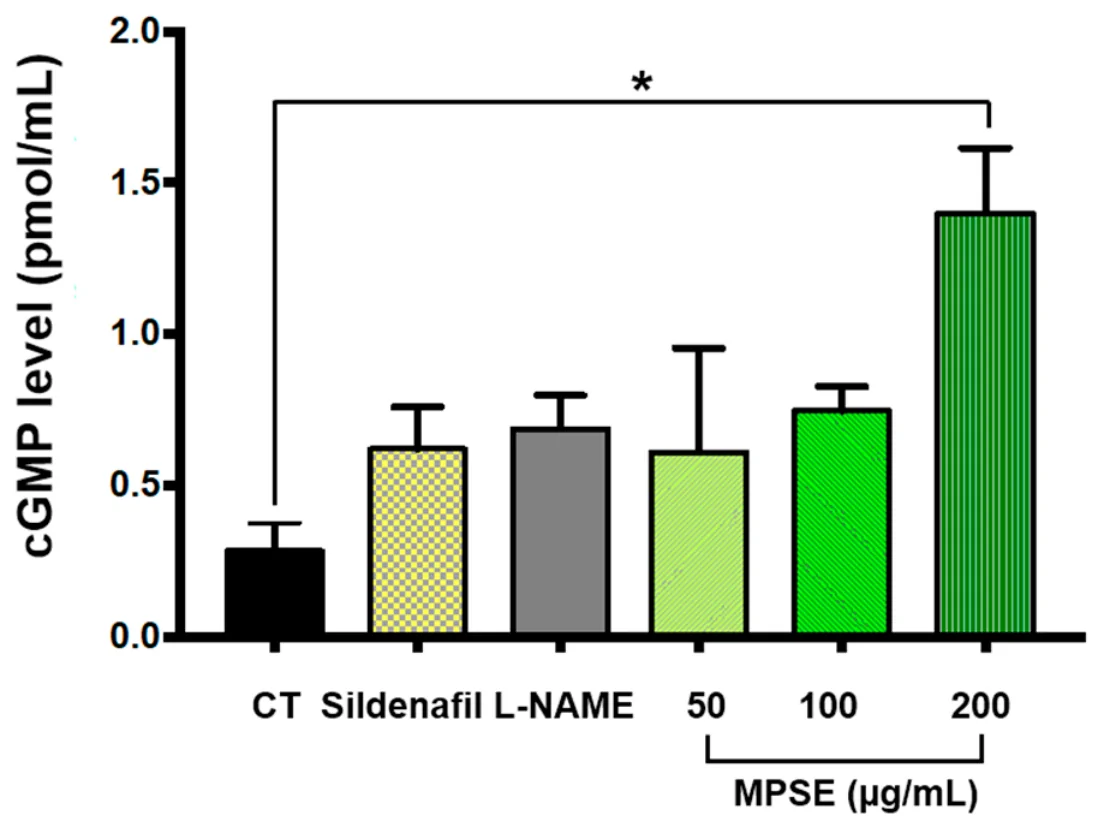
Effect of MPSE on intracellular cGMP levels in PASMCs. Intracellular cGMP levels were determined using a competitive ELISA assay. Data are presented as mean ± S.E.M. (*n* = 3). Groups were compared by one-way ANOVA followed by Tukey’s multiple comparison test. * *p* < 0.05 indicates a statistically significant difference between the indicated groups. CT, control group.

MPSE at a concentration of 200 µg/mL significantly increased intracellular cGMP levels in PASMCs compared with the control group. In contrast, MPSE at concentrations of 50 and 100 µg/mL, sildenafil, and L-NAME did not significantly alter intracellular cGMP levels compared with the control group (Figure 6).

#### 3.2.5. Effects of MPSE on PDE5 Inhibitory Activity

**Figure 7:**
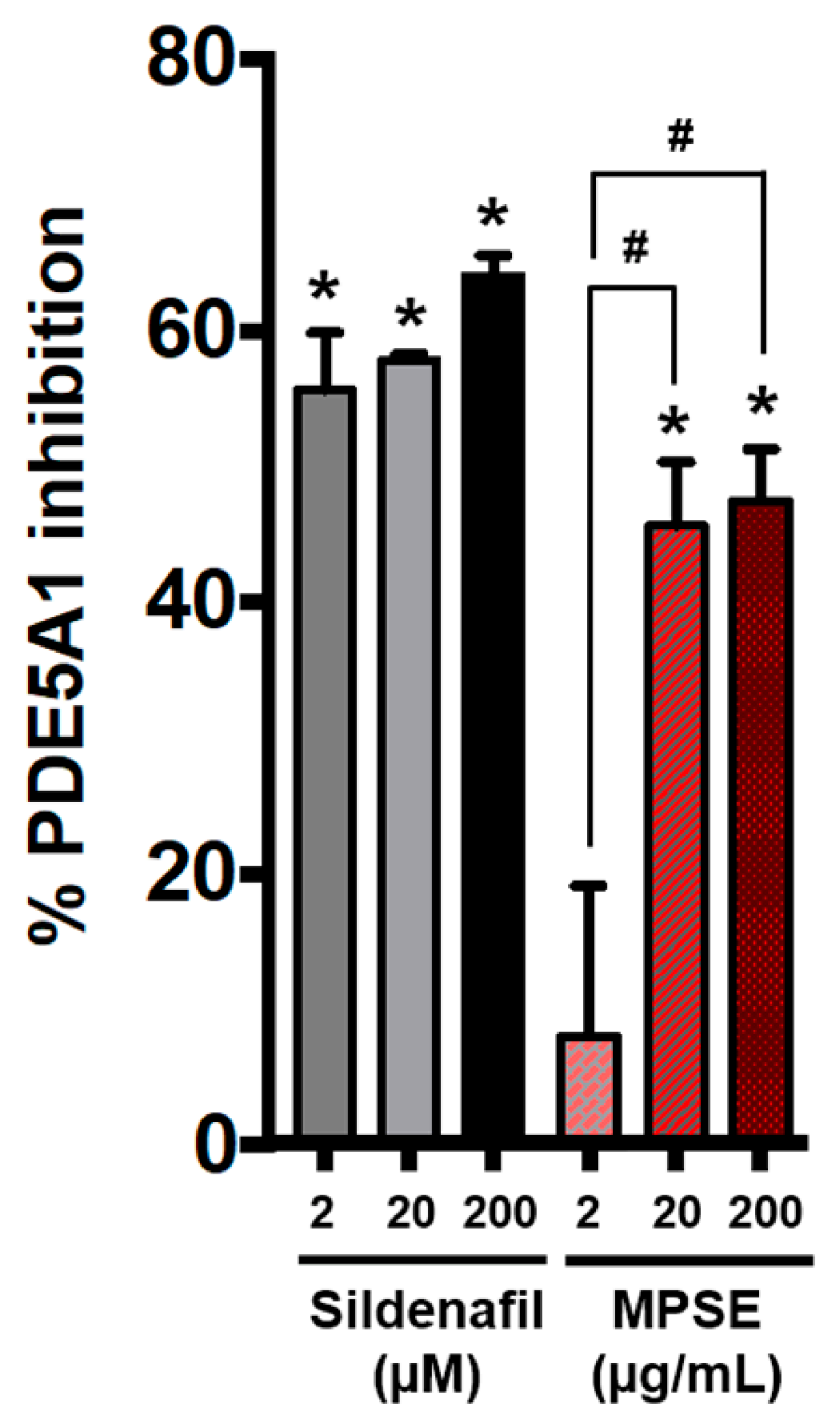
Effect of MPSE on PDE5A1 inhibitory activity. PDE5A1 inhibition was determined using a fluorescence polarization–based assay and is expressed as percentage inhibition relative to the pooled mean signal of the buffer-only control (negative control). The negative control therefore has a mean of approximately 0% inhibition by definition and is not displayed in the graph, although it was retained as a group in the statistical analysis. Data are presented as mean ± S.E.M. (*n* = 3). MPSE and sildenafil were analyzed separately, each together with the negative control, by one-way ANOVA followed by Tukey’s multiple comparison test. * *p* < 0.05 compared with the negative control. ^#^ *p* < 0.05 between the indicated concentrations.

MPSE significantly inhibited PDE5A1 activity at 20 and 200 µg/mL compared with the negative control, whereas inhibition at 2 µg/mL did not differ significantly from the negative control. Inhibition at 20 and 200 µg/mL was significantly greater than at 2 µg/mL but did not differ between these two concentrations. Sildenafil, used as a positive control inhibitor, significantly inhibited PDE5A1 activity at all tested concentrations, with no significant differences among the three concentrations (Figure 7).

### 3.3. In Vivo Evaluation of MPSE on Mounting Behavior and Safety Profile

#### 3.3.1. Effects of MPSE on Mounting Frequency and Serum Testosterone Levels in Male Rats

Mounting frequency was assessed before treatment (Day 0) and on Days 5, 10, and 15, and within-group changes over time are presented in Table 1. In the control group, mounting frequency decreased significantly on Day 5 compared with Day 0, increased significantly on Day 10 relative to Day 5 while remaining significantly below Day 0, and decreased again on Day 15 to a level significantly below all preceding time points. In male rats treated with sildenafil, mounting frequency was significantly increased on Days 5 and 10 compared with Day 0, but significantly decreased on Day 15 relative to all preceding time points. In contrast, MPSE at 50 mg/kg significantly increased mounting frequency only on Day 15, with no significant changes on Days 5 or 10. MPSE at 100 mg/kg significantly increased mounting frequency on Day 10 compared with Days 0 and 5, and values on Day 15 remained significantly higher than Days 0 and 5 despite a significant decline from Day 10. MPSE at 200 mg/kg produced an earlier response, with significant increases on both Days 5 and 10 compared with Day 0, and mounting frequency on Day 15 remained significantly higher than Day 0 despite a significant decline from Day 10.

**Figure 8:**
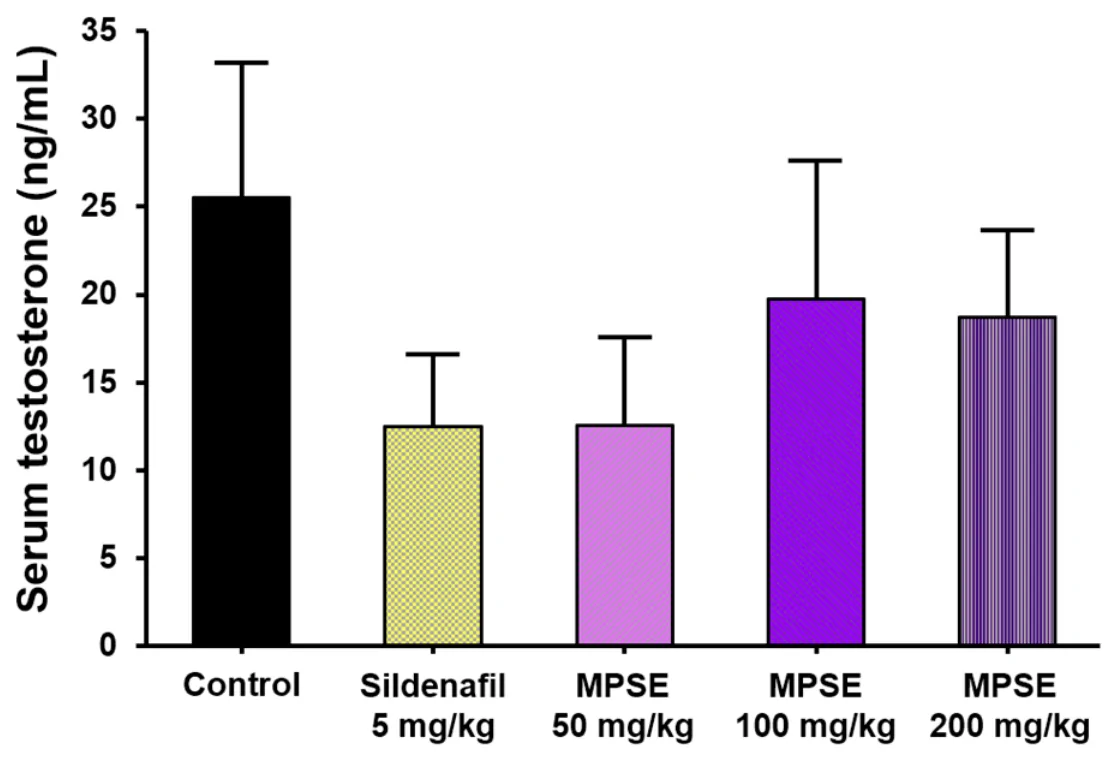
Effects of MPSE on serum testosterone levels in male rats. Data are presented as mean ± S.E.M. (*n* = 6). Serum testosterone was measured on Day 15 and compared among groups using one-way ANOVA followed by Tukey’s multiple comparison test.

At the end of the experimental period (Day 15), serum testosterone levels were measured in all groups. No significant differences were observed in male rats treated with sildenafil or MPSE at 50, 100, and 200 mg/kg compared with the control group (Figure 8).

#### 3.3.2. Effects of MPSE on Anxiety-like Behavior in Male Rats

The effects of MPSE on anxiety-like behavior in male rats were evaluated using the elevated plus maze test, and the results are presented in Table 2. Male rats treated with sildenafil showed a significant decrease in closed-arm time compared with the control group. Meanwhile, male rats treated with diazepam exhibited a significant increase in open-arm time and a significant decrease in closed-arm time compared with the control group. In contrast, treatment with MPSE at doses of 50, 100, and 200 mg/kg did not significantly alter either open-arm time or closed-arm time compared with the control group. In addition, the percentage of time spent in the open arms was higher in male rats treated with sildenafil (16.75%) and diazepam (25.22%) than in the control group (5.94%). However, male rats treated with MPSE at all tested doses showed lower percentages of time spent in the open arms than the control group.

#### 3.3.3. Effects of MPSE on Locomotor Activity in Male Rats

**Figure 9:**
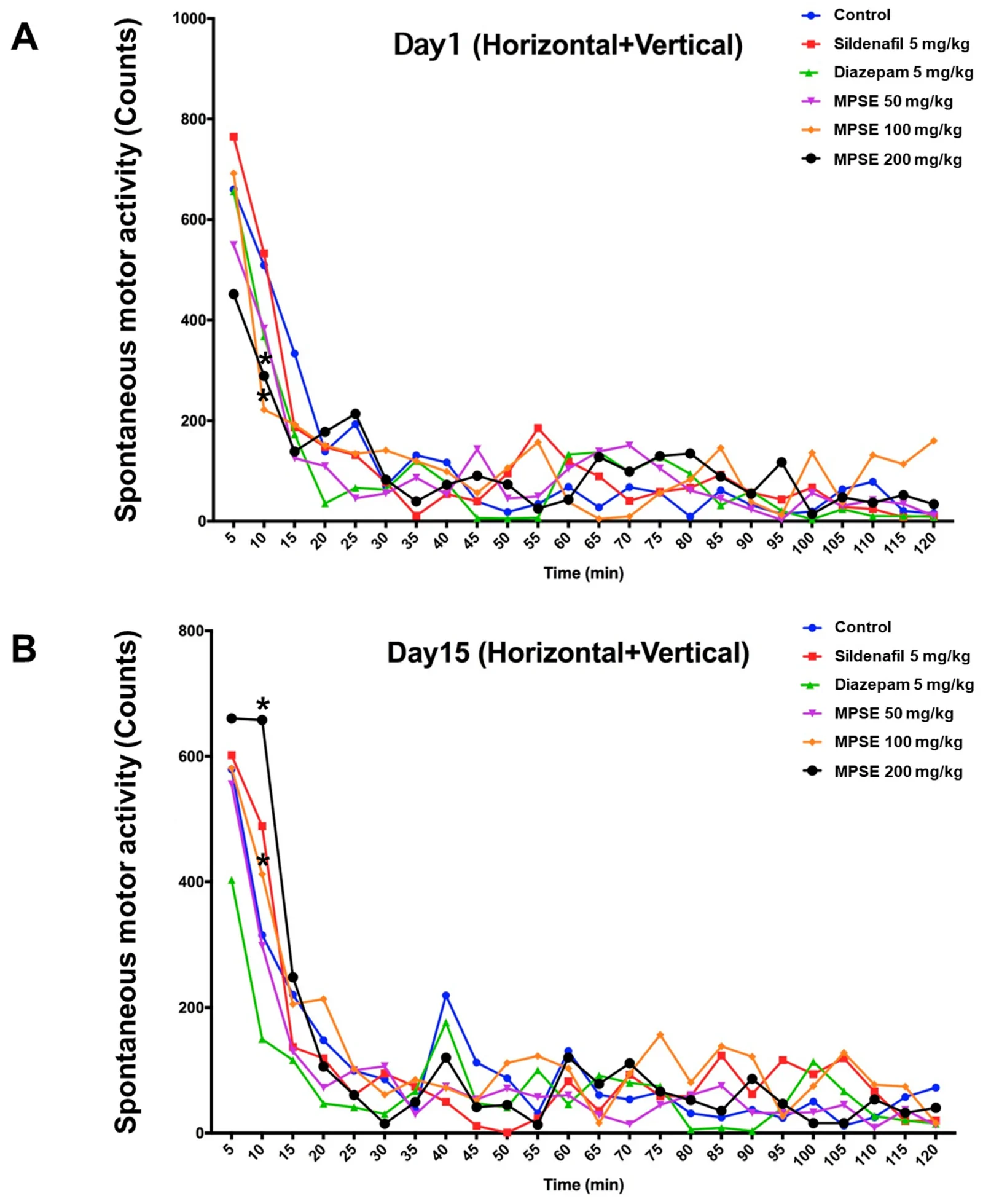
Effects of MPSE on locomotor activity in male rats on Days 1 (**A**) and 15 (**B**). Data are presented as mean ± S.E.M. (*n* = 6). Groups were compared at each 5-min observation interval by one-way ANOVA followed by Tukey’s multiple comparison test. * *p* < 0.05 compared with the control group.

Locomotor activity (horizontal + vertical counts) was evaluated beginning 30 min after treatment administration on Day 1. At 10 min of observation (40 min post-treatment), male rats treated with MPSE at doses of 100 and 200 mg/kg showed significantly lower locomotor activity compared with the control group. No significant differences among the groups were observed during the remaining observation period. In addition, locomotor activity in male rats treated with sildenafil or diazepam did not differ significantly from that of the control group throughout the 120-min observation period (Figure 9A). After 15 days of daily administration, locomotor activity was reassessed on Day 15. At 10 min of observation (40 min post-treatment), male rats treated with MPSE at doses of 100 and 200 mg/kg exhibited significantly higher locomotor activity compared with the control group; however, after this time point, no significant differences from the control group were observed during the subsequent observation period. No significant differences were detected between the sildenafil- or diazepam-treated groups and the control group at any time point (Figure 9B).

#### 3.3.4. Effects of MPSE on Mean Arterial Pressure and Heart Rate in Normotensive Male Rats

The effects of MPSE on mean arterial pressure and heart rate in normotensive male rats are presented in Table 3. Before treatment, there were no significant differences in mean arterial pressure or heart rate among the experimental groups. Thirty minutes after treatment administration, sildenafil and MPSE at doses of 50, 100, and 200 mg/kg body weight did not significantly alter mean arterial pressure or heart rate compared with either their respective values before treatment or the control group after treatment. In contrast, prazosin significantly decreased mean arterial pressure and significantly increased heart rate compared with both their respective values before treatment and the control group after treatment.

#### 3.3.5. Effects of MPSE on Mean Arterial Pressure and Heart Rate in L-NAME-Induced Hypertensive Male Rats

The effects of MPSE on mean arterial pressure and heart rate in L-NAME-induced hypertensive male rats are shown in Table 4. Before treatment administration, mean arterial pressure and heart rate did not differ significantly among the experimental groups. At 30 min after treatment, sildenafil and MPSE at doses of 50, 100, and 200 mg/kg did not significantly change mean arterial pressure or heart rate compared with their respective pretreatment values or with the control group after treatment. In contrast, prazosin significantly reduced mean arterial pressure compared with both its corresponding pretreatment value and the control group after treatment. Heart rate in the prazosin-treated group did not differ significantly from its pretreatment value or from the control group after treatment.

## 4. Discussion

### 4.1. Standardization of MPSE and Its Chemical Markers

Erectile dysfunction is closely associated with endothelial dysfunction and impairment of the NO–cGMP–PDE5 pathway, which play a central role in penile erection [12,13,15]. Although PDE5 inhibitors are considered first-line therapy, their use may be limited by contraindications, adverse effects, and treatment costs, highlighting the need for alternative therapeutic options [5,22,24]. *M. pruriens* var. *pruriens* has traditionally been used to enhance male sexual function and has demonstrated beneficial effects on reproductive parameters in experimental studies [26,28,29]. Several studies have reported that different phytochemical constituents and extract preparations of *M. pruriens* exhibit pharmacological activities relevant to sexual function, behavioral regulation, and cardiovascular control. A polar extract rich in catechol compounds and polyphenols has been shown to enhance eNOS gene expression and increase intracellular cGMP levels in vitro, together with improved mounting behavior in vivo [34]. Similarly, both an ethanolic seed extract containing L-DOPA and an aqueous seed extract of *M. pruriens* have been reported to significantly increase mounting frequency in experimental animals [35,36]. Beyond its effects on sexual behavior, an aqueous seed extract of *M. pruriens*, which contains L-DOPA, was reported to reduce anxiety-like behavior in an ethanol withdrawal syndrome model in mice [37]. In addition, protein hydrolysates derived from *M. pruriens* seeds have demonstrated antihypertensive activity in vivo, likely through angiotensin-converting enzyme-inhibitory mechanisms [38]. Standardizing *M. pruriens* var. *pruriens* seed extract is essential for pharmacological consistency and safety, particularly because acute gastrointestinal and neurological toxicity has been reported following ingestion of raw seeds, an outcome attributed to their high and variable L-DOPA content [39]. Consistent quantification of this constituent is therefore a prerequisite for pharmacological evaluation. Our previous study established the safety of the ethanolic seed extract, in which a single oral dose of 5000 mg/kg body weight and repeated oral administration at 100, 500, and 2500 mg/kg body weight daily for 270 days produced no abnormal clinical signs or toxicological alterations in rats, including the female reproductive organs (ovaries and uterus) and the male reproductive organs (testes and epididymides), which showed no gross or histopathological abnormalities [33]. Building on this evidence, the present study evaluated the pharmacological activity of the standardized extract in vitro and in vivo, as well as its cardiovascular safety, since hemodynamic effects are an important limitation of current pharmacological treatments for erectile dysfunction [5,22]. In the present study, roasted seed parameters met preliminary specifications, reflecting effective post-harvest handling and low inorganic contamination. Chemical profiling confirmed L-DOPA as the main constituent, and HPTLC analysis (Figure 1) measured L-DOPA at 1.39 mg/mL (Rf 0.38). An additional unidentified spot at Rf 0.42 (UV 366 nm) suggests a complex chemical matrix that may contribute to synergistic effects.

### 4.2. Effects of MPSE on eNOS Expression and NO Production in HUVECs

Endothelial dysfunction is recognized as a central pathophysiological mechanism underlying erectile dysfunction because impaired NO bioavailability reduces cavernosal smooth muscle relaxation and penile blood inflow [5,40]. In penile tissue, eNOS plays a critical role in erectile physiology by generating NO in response to endothelial stimulation, which subsequently activates soluble guanylyl cyclase in cavernosal smooth muscle cells and promotes vasodilation [41,42]. To investigate these mechanisms, HUVECs were selected to represent vascular endothelial cells, and PASMCs were selected to represent vascular smooth muscle cells. These primary human cell models share the NO/cGMP signaling pathway that physiologically mediates cavernosal smooth muscle relaxation and penile erection, and therefore provide a mechanistically appropriate in vitro system for evaluating compounds with potential erectogenic activity [12,13,43,44]. In the present study, L-NAME and sildenafil were included as mechanistic controls to verify the specificity and validity of the in vitro experimental system. The results showed that L-NAME did not significantly alter eNOS gene expression compared with the control group, which is consistent with its established role as a competitive inhibitor of NOS enzymatic activity rather than a regulator of NOS3 transcription [45]. Similarly, sildenafil served to further support pathway specificity, as its pharmacological action is mediated through inhibition of PDE5 rather than stimulation of eNOS gene expression [14,21]. The absence of changes in eNOS expression following treatment with these pathway-specific modulators indicates that the experimental system did not produce nonspecific or false-positive responses. Importantly, MPSE at all tested concentrations significantly increased eNOS gene expression in HUVECs, indicating a stimulatory effect on endothelial NO synthesis. NO is the principal mediator of penile erection and plays a central role in regulating cavernosal smooth muscle relaxation through activation of the NO–cGMP pathway. In penile tissue, NO is synthesized primarily by eNOS in endothelial cells and by neuronal nitric oxide synthase (nNOS) in nitrergic nerves during sexual stimulation [41,46]. The released NO diffuses into adjacent cavernosal smooth muscle cells, where it activates soluble guanylyl cyclase and increases intracellular cGMP levels. This signaling cascade reduces cytosolic calcium concentrations, resulting in smooth muscle relaxation, dilation of penile arteries, and increased cavernosal blood inflow required for penile erection [47]. As expected, NO production in the L-NAME-treated group remained lower, consistent with its role as a NOS inhibitor [45], whereas sildenafil did not significantly alter NO levels compared with the control group, reflecting its downstream mechanism of action that primarily prevents cGMP degradation rather than increasing NO synthesis [14,21]. In the present study, MPSE significantly increased NO production in HUVECs at the concentration of 200 µg/mL compared with the control group, indicating enhanced endothelial NO bioavailability. This observation is consistent with previous in vitro evidence demonstrating that both the crude extract and the polarity fractions prepared from *M. pruriens* seeds enhance NO production in endothelial cells through upregulation of eNOS expression [34].

### 4.3. Effects of MPSE on Intracellular cGMP in PASMCs and PDE5A1 Enzyme Activity

Following NO release, activation of soluble guanylyl cyclase leads to increased intracellular cGMP, a key second messenger responsible for regulating cavernosal smooth muscle relaxation and facilitating penile erection [46]. L-NAME acts upstream by inhibiting NOS, thereby reducing NO availability and limiting subsequent activation of soluble guanylyl cyclase and downstream cGMP formation, whereas sildenafil acts downstream by preventing cGMP degradation [14,21]. In the present study, neither treatment significantly altered intracellular cGMP levels compared with the control group. This may reflect the limited basal NO–cGMP signaling in PASMCs under monoculture conditions, in the absence of endothelial cells or nitrergic nerve-derived NO. Under these conditions, inhibition of NOS has little activity to suppress, and inhibition of cGMP degradation has little ongoing synthesis to preserve. Notably, MPSE at 200 µg/mL significantly increased intracellular cGMP levels in PASMCs despite these conditions, which may indicate that the extract, containing numerous other constituents, raises cGMP through mechanisms other than inhibition of its degradation. To date, evidence demonstrating the direct effect of *M. pruriens* seed extract on intracellular cGMP levels in vascular smooth muscle cells remains limited. The present findings provide additional cellular evidence supporting downstream activation of the NO–cGMP pathway in smooth muscle cells. Intracellular cGMP concentrations are tightly regulated by PDE5, which hydrolyzes cGMP to the inactive 5′-GMP, thereby terminating erection-related signaling [14,48]. As expected, sildenafil produced significant inhibition of PDE5A1 enzymatic activity at all tested concentrations, with a similar magnitude across the concentration range, consistent with its well-established role as a selective PDE5 inhibitor and its reported low-nanomolar potency [14,21], thereby supporting the validity of the assay system used to evaluate MPSE. Interestingly, MPSE directly inhibited PDE5A1 enzymatic activity in an in vitro enzyme assay, indicating its ability to interfere with enzymatic cGMP degradation. The inhibition of PDE5A1 by MPSE, particularly at concentrations of 20 and 200 µg/mL, suggests that the extract may prolong cGMP-mediated vasorelaxation by limiting its enzymatic degradation, thereby supporting sustained cavernosal smooth muscle relaxation. Similar PDE5 inhibitory activity has also been reported for methanolic extracts of *M*. *pruriens* seeds in previous in vitro enzyme-based assays [49]. Taken together, the in vitro assays reveal the mechanism of action through the NO–cGMP–PDE5 pathway. MPSE acted on this pathway by increasing eNOS expression, enhancing NO production, elevating intracellular cGMP levels, and inhibiting PDE5A1 enzymatic activity. However, these findings were obtained from in vitro cell-based assays, which cannot fully explain the responses occurring in the whole body. Therefore, an in vivo study was conducted to clarify how MPSE acts to enhance sexual performance and whether it is sufficiently safe to be developed into a herbal product for future use in humans.

### 4.4. Effects of MPSE on Mounting Frequency and Serum Testosterone in Male Rats

Mounting frequency is widely used as an indicator of sexual motivation and copulatory performance in male rodents and is influenced by androgenic status, particularly circulating testosterone levels [50]. However, mounting behavior can also occur independently of systemic testosterone changes, as the onset of copulatory activity has been reported in male rats without preceding alterations in circulating testosterone levels [51]. Importantly, adequate penile erection is required for effective copulatory performance, as erectile responses facilitate pelvic thrusting efficiency and progression from mounting to intromission [52]. Therefore, enhancement of erection-related signaling, particularly through activation of the NO–cGMP pathway, may contribute to increased mounting behavior even in the absence of changes in circulating testosterone levels [53]. In the current study, ovariectomized, hormone-primed female rats were used to standardize sexual receptivity and minimize variability associated with endogenous estrous cycle fluctuations, thereby allowing mounting frequency to serve as a reliable indicator of male sexual motivation and copulatory performance independent of differences in female receptivity [54]. All groups were subjected to the same repeated-testing schedule. Sustained copulatory activity induces a transient state of reduced sexual motivation in male rats [52]. Consistent with this, mounting frequency in the control group was significantly lower on Day 15 than the baseline value, indicating a reduction in copulatory performance associated with the testing schedule itself rather than with treatment. Sildenafil significantly increased mounting frequency on Days 5 and 10 compared with baseline, confirming that the behavioral model was responsive to pharmacological facilitation of the NO–cGMP–PDE5 pathway. However, mounting frequency in the sildenafil group on Day 15 also fell below the baseline value. This finding is consistent with the mechanism of sildenafil as a selective PDE5 inhibitor, which prevents cGMP degradation but does not increase NO production [14,21]. Because the animals used in the present study were sexually active and had an intact NO–cGMP–PDE5 pathway, inhibition of cGMP degradation alone was unlikely to produce an effect that was maintained throughout repeated administration. In contrast, MPSE increased eNOS expression, NO production, and intracellular cGMP levels in addition to inhibiting PDE5A1 activity in vitro. These findings indicate that MPSE acts through more diverse mechanisms than sildenafil. Consistent with this, mounting frequency on Day 15 remained significantly higher than baseline at all three doses. Moreover, these increases occurred without any change in serum testosterone levels, suggesting that the enhancement of mounting behavior was mediated through vascular rather than androgenic mechanisms. This is consistent with previous reports that both ethanolic and aqueous seed extracts of *M. pruriens* have been shown to increase mounting frequency and other copulatory parameters in experimental animals [29,35], which is in agreement with the mounting behavioral findings observed in the present study. However, in contrast to the present results, those studies also demonstrated significant elevations in serum testosterone levels following treatment. This difference may be explained by the longer treatment durations used in previous investigations, which were likely sufficient to modulate hypothalamic–pituitary–gonadal axis activity and stimulate steroidogenesis [55]. In comparison, the shorter treatment period used in the present study may account for the absence of detectable changes in circulating testosterone despite the observed increase in mounting frequency. Notably, the absence of increased serum testosterone levels following MPSE administration may represent a safety advantage, since excessive testosterone exposure has been associated with erythrocytosis and increased risks of thromboembolic and cardiovascular complications [56,57].

### 4.5. Effects of MPSE on Anxiety-like Behavior and Locomotor Activity in Male Rats

To further clarify whether the enhancement of mounting frequency observed in the present study was associated with central nervous system stimulation, anxiety-like behavior, and locomotor activity were evaluated. Anxiety has been reported to impair erectile function by enhancing sympathetic nervous system activity and altering central regulatory mechanisms involved in sexual arousal, thereby interfering with normal erectile responses [18,19]. The elevated plus maze test is a widely used behavioral model for assessing anxiety-like behavior in rodents based on their natural avoidance of open and elevated spaces. Because anxiety can interfere with sexual arousal and erectile responses through central neural mechanisms, this test was performed to determine whether the observed increase in mounting behavior was associated with alterations in anxiety-related activity [58]. In the present study, diazepam, used as an anxiolytic positive control, significantly increased open-arm time, confirming the responsiveness of the behavioral model to centrally acting anxiolytic agents [58], whereas sildenafil produced changes in arm exploration without evidence of a clear anxiolytic effect. Similarly, MPSE did not significantly alter open-arm or closed-arm time in the elevated plus maze test, indicating the absence of anxiolytic or anxiogenic effects. Increased locomotor activity has been reported to reflect enhanced central nervous system activation and exploratory behavior, which may indirectly facilitate the expression of male sexual behavior by increasing responsiveness to receptive females and the probability of mounting behavior [59]. Therefore, spontaneous locomotor activity was assessed using the open field test by quantifying horizontal and vertical movements as indices of general motor activity and exploratory drive [60]. This assessment allowed discrimination between specific facilitation of sexual performance and nonspecific central excitatory effects that could secondarily influence mounting frequency. Because responses to centrally acting agents change over time, locomotor activity was examined both at individual observation intervals and as the summed counts within successive 30-min intervals, allowing transient changes to be distinguished from effects sustained across a broader time window [59]. Under this analysis, diazepam (5 mg/kg) did not significantly reduce locomotor activity at any time point following either acute or repeated administration. This is consistent with a previous report in which oral administration of diazepam at 15 mg/kg did not significantly alter elapsed time to probe contact, an index of exploratory locomotor activity, at either 30 or 60 min after gavage [61]. The absence of marked locomotor suppression in the present study may also be attributable to the oral route of administration, as more pronounced reductions in locomotor activity have been reported following parenteral administration of diazepam, including intravenous dosing, which produces more rapid central nervous system depressant effects in rodents [62,63]. As for sildenafil, it did not alter locomotor activity throughout the observation period, consistent with its peripheral mechanism of action as a selective PDE5 inhibitor acting on penile vascular tissue rather than on central pathways [14,21]. Meanwhile, MPSE did not alter locomotor activity when the data were analyzed by summed intervals. Therefore, the increase in mounting frequency observed with MPSE was not attributable to changes in locomotor activity. In line with these findings, a previous behavioral study reported that administration of a hydroalcoholic extract of *M. pruriens* did not significantly modify time spent in the center or closed arms in healthy rats, supporting the interpretation that *M. pruriens* produces minimal central behavioral modulation under normal physiological conditions [64].

### 4.6. Effects of MPSE on Mean Arterial Pressure and Heart Rate in Normotensive and Hypertensive Male Rats

To evaluate the cardiovascular safety of MPSE, potential hemodynamic side effects were assessed under both normotensive and hypertensive conditions. In the present study, prazosin, sildenafil, and MPSE were first administered to normotensive rats in order to determine whether these treatments produced acute alterations in arterial pressure or heart rate under normal physiological conditions. The results demonstrated that prazosin markedly reduced mean arterial pressure and increased heart rate, consistent with its mechanism as an α_1_-adrenergic receptor antagonist that decreases peripheral vascular resistance and induces reflex tachycardia [65]. In contrast, sildenafil and MPSE did not significantly alter mean arterial pressure or heart rate in normotensive rats, indicating that neither treatment produced acute hypotensive effects or compensatory tachycardia under baseline conditions. Because hypertension is one of the most common cardiovascular comorbidities associated with erectile dysfunction, and approximately 81.6% of hypertensive men have been reported to exhibit erectile dysfunction [66], evaluation under hypertensive conditions is clinically relevant. Moreover, PDE5 inhibitors enhance erectile responses through NO–cGMP-mediated vasodilation, and their concomitant use with antihypertensive drugs may increase the risk of treatment-related hypotension due to additive blood pressure–lowering effects [5]. Therefore, in the present study, hypertension was experimentally induced using L-NAME prior to administration of prazosin, sildenafil, and MPSE, in order to assess whether these agents altered mean arterial pressure or heart rate in hypertensive rats. Under hypertensive conditions, prazosin significantly reduced arterial pressure without a significant change in heart rate, consistent with its peripheral vasodilatory action. Sildenafil at 5 mg/kg did not significantly alter mean arterial pressure or heart rate in hypertensive rats. This is consistent with a previous report in which sildenafil at 1, 3, and 10 mg/kg produced no significant hemodynamic change, whereas a significant reduction in mean arterial pressure was observed only at 30 mg/kg [67]. In contrast, MPSE at 50, 100, and 200 mg/kg did not significantly alter mean arterial pressure or heart rate in hypertensive rats. These findings indicate that MPSE did not produce acute hemodynamic effects in hypertensive rats under the conditions of the acute L-NAME model.

### 4.7. Integration of Mechanisms and Study Limitations

In general, increased mounting can arise through several mechanisms. These include peripheral erectile function via the NO–cGMP pathway [34], androgenic testosterone signaling [50,51], psychogenic and arousal-related factors [17,19], and central dopaminergic activity [50,53]. In the present study, MPSE upregulated eNOS expression, increased NO production and intracellular cGMP levels, and inhibited PDE5A1 activity in vitro. In addition, MPSE increased mounting frequency relative to baseline without altering serum testosterone or anxiety-related behavior, and without producing a sustained change in locomotor activity. These findings indicate that androgenic and psychogenic or arousal-related mechanisms are unlikely to account for the increase in mounting, which is therefore more likely attributable to peripheral vascular mechanisms underlying erectile function. Together with the absence of significant changes in mean arterial pressure and heart rate, this suggests that MPSE may warrant evaluation as an alternative or adjunctive option in patients with hypertension. In addition to the findings of the present study, four limitations should be acknowledged. First, the MPSE was standardized using L-DOPA as a chemical marker. Further analysis of the phytochemical profile of the 80% ethanolic extract to identify the specific constituent responsible for its pharmacological activity would therefore allow a more biologically relevant marker to be established for future standardization. Second, erectile function was not assessed directly; confirmation would require measurement of intracavernosal pressure, complemented by ex vivo vasorelaxation studies in isolated dorsal penile artery preparations. Third, the effect of MPSE on central neurotransmitter systems involved in sexual behavior, such as dopaminergic signaling, was not directly assessed. Fourth, the cardiovascular safety assessment relied on an acute L-NAME model, which reproduces a nitric oxide–deficient pressor state rather than the chronic vascular changes present in patients with long-standing hypertension, and did not include co-administration of MPSE or sildenafil with antihypertensive agents, which is clinically relevant given the frequent coexistence of hypertension and erectile dysfunction. Further studies addressing these aspects would provide greater clarity regarding the pharmacological activity and safety profile of MPSE.

## 5. Conclusions

The present study demonstrates that MPSE modulates erectile function-related mechanisms through coordinated effects on the NO–cGMP–PDE5 pathway. The extract increased eNOS expression, NO production, and intracellular cGMP levels, and inhibited PDE5 activity, consistent with the observed increase in mounting frequency relative to baseline in male rats without changes in serum testosterone levels, anxiety-like behavior, or locomotor activity, indicating a predominantly peripheral vascular mechanism of action. Importantly, MPSE did not affect mean arterial pressure or heart rate, supporting its favorable central nervous system and cardiovascular safety profile. Collectively, by integrating vascular-cell, hormonal, behavioral, and cardiovascular findings for a single standardized extract, aspects previously examined separately, these findings identify MPSE as a promising plant-derived candidate that warrants further investigation for the management of erectile dysfunction.

## Figures and Tables

**Table 1:** Effects of MPSE on mounting frequency in male rats.

Mounting Frequency (Times)	Group				
	Control	Sildenafil 5 mg/kg	MPSE 50 mg/kg	MPSE 100 mg/kg	MPSE 200 mg/kg
**Day 0**	19.2 ± 4.9 ^bcd^	17.2 ± 4.5 ^bcd^	21.8 ± 2.9 ^d^	21.8 ± 4.7 ^cd^	13.8 ± 4.1 ^bcd^
**Day 5**	13.3 ± 2.9 ^acd^	22.3 ± 3.2 ^ad^	20.2 ± 5.1 ^d^	22.3 ± 3.6 ^cd^	21.0 ± 3.8 ^ac^
**Day 10**	17.8 ± 3.1 ^abd^	22.8 ± 6.2 ^ad^	21.0 ± 3.1 ^d^	29.7 ± 6.2 ^abd^	25.7 ± 3.9 ^abd^
**Day 15**	11.5 ± 1.7 ^abc^	12.0 ± 1.8 ^abc^	23.7 ± 4.8 ^abc^	25.8 ± 6.5 ^abc^	22.3 ± 6.4 ^ac^

Data are presented as mean ± S.E.M. (*n* = 6). Mounting frequency across assessment days within each group was analyzed using a linear mixed model followed by a Bonferroni post hoc test. ^a^ *p* < 0.05 compared with Day 0. ^b^ *p* < 0.05 compared with Day 5. ^c^ *p* < 0.05 compared with Day 10. ^d^ *p* < 0.05 compared with Day 15.

**Table 2:** Effects of MPSE on time spent in the open and closed arms of the elevated plus maze in male rats.

Group	Open-Arm Time (s)	Closed-Arm Time (s)	Open-Arm Time/Total Time 300 s (%)
Control	17.83 ± 12.68	250.25 ± 21.70	5.94
Sildenafil 5 mg/kg	50.25 ± 22.46	144.67 ± 50.72 *	16.75
Diazepam 5 mg/kg	75.67 ± 18.37 *	127.83 ± 13.66 *	25.22
MPSE 50 mg/kg	1.00 ± 1.00	221.67 ± 39.10	0.33
MPSE 100 mg/kg	8.17 ± 5.43	211.33 ± 40.27	2.72
MPSE 200 mg/kg	2.00 ± 2.00	258.50 ± 17.27	0.67

Data are presented as mean ± S.E.M. (*n* = 6). Open-arm and closed-arm times were compared among groups by the Kruskal–Wallis test followed by Dunn’s multiple comparison test. * *p* < 0.05 compared with the control group.

**Table 3:** Effects of MPSE on mean arterial pressure and heart rate in normotensive male rats.

Group	Before Treatment		After Treatment	
	Mean Arterial Pressure (mmHg)	Heart Rate (Beat/min)	Mean Arterial Pressure (mmHg)	Heart Rate (Beat/min)
Control	112.67 ± 4.13	345.06 ± 18.48	110.00 ± 4.55	347.67 ± 18.88
Sildenafil 5 mg/kg	108.56 ± 3.76	357.78 ± 21.13	109.78 ± 5.05	358.00 ± 24.94
Prazosin 2 mg/kg	111.95 ± 8.24	358.95 ± 11.74	99.39 ± 0.28 *^#^	398.83 ± 15.23 *^#^
MPSE 50 mg/kg	101.95 ± 2.55	355.31 ± 16.43	113.19 ± 4.99	357.92 ± 16.79
MPSE 100 mg/kg	107.61 ± 4.61	343.67 ± 15.21	108.22 ± 4.00	336.45 ± 12.26
MPSE 200 mg/kg	111.06 ± 4.58	338.11 ± 5.89	102.11 ± 1.14	335.78 ± 20.68

Data are presented as mean ± S.E.M. (*n* = 6). Between-group comparisons at each time point were made by one-way ANOVA followed by Tukey’s multiple comparison test, and within-group comparisons of values before and after treatment were made by the paired *t*-test. * *p* < 0.05 compared with the control group; ^#^ *p* < 0.05 compared with the values before treatment.

**Table 4:** Effects of MPSE on mean arterial pressure and heart rate in hypertensive male rats.

Group	Before Treatment		After Treatment	
	Mean Arterial Pressure (mmHg)	Heart Rate (Beat/min)	Mean Arterial Pressure (mmHg)	Heart Rate (Beat/min)
Control	145.17 ± 5.31	353.39 ± 10.70	151.39 ± 4.27	324.83 ± 12.75
Sildenafil 5 mg/kg	136.39 ± 9.65	341.56 ± 10.03	140.06 ± 8.10	363.56 ± 24.93
Prazosin 2 mg/kg	145.61 ± 4.75	325.72 ± 8.15	118.56 ± 4.47 *^#^	359.39 ± 14.89
MPSE 50 mg/kg	142.17 ± 3.91	331.61 ± 8.03	138.22 ± 4.03	306.45 ± 10.46
MPSE 100 mg/kg	139.67 ± 4.71	350.89 ± 17.59	141.20 ± 4.79	343.42 ± 11.29
MPSE 200 mg/kg	139.44 ± 3.67	329.95 ± 20.45	141.59 ± 3.97	339.17 ± 12.92

Data are presented as mean ± S.E.M. (*n* = 6). Between-group comparisons at each time point were made by one-way ANOVA followed by Tukey’s multiple comparison test, and within-group comparisons of values before and after treatment were made by the paired *t*-test. * *p* < 0.05 compared with the control group; ^#^ *p* < 0.05 compared with the values before treatment.

## Data Availability

The original contributions presented in this study are included in the article. Further inquiries can be directed to the corresponding author.
